# Innovation in physical education: The role of cognitive factors and self-efficacy

**DOI:** 10.3389/fpsyg.2022.959979

**Published:** 2022-08-10

**Authors:** Songpu Li, Ruilin Xu, Zijian Zhao

**Affiliations:** School of Physical Education (Main Campus), Zhengzhou University, Zhengzhou, China

**Keywords:** mental workload, self-efficacy, decision-making process, intention, bright sides

## Abstract

Among the beliefs related to teaching work, self-efficacy stands out and encourage innovation across the global education systems. Specifically, the lack of interest among instructors in introducing innovative techniques in physical education is a concern across China. Therefore, this study intends to investigate the role of cognitive indicators (mental workload, decision-making process, innovation in physical education, and self-efficacy) of innovation in physical education across China. This study opted for quantitative techniques, including using a structured questionnaire to collect data from targeted respondents through the survey techniques. Moreover, 800 questionnaires were circulated, and as a result, 420 usable responses were attained, making the overall response rate stand at 40%. The results indicate that the above-stated cognitive factors, along with self-efficacy, have a positive role in causing innovation across the physical education exchequer of China. Likewise, self-efficacy played the mediating role between cognitive indicators and innovation in physical education in China. The study has notable theoretical and practical implications for the policymakers in terms of introducing policies that could help increase the cognitive state of educationists, which in turn possibly will help make them pursue innovation within the education system of China.

## Introduction

It is of great importance to understand that physical education plays an enormous role in shaping the thought patterns of individuals by providing them with an opportunity to learn. Physical education has existed for ages, but the most critical concern is the scarcity of the provision for introducing innovation within it (Mitrotasios et al., 2022). Indeed, as society grows, the issues related to physical education are getting complicated. In China, the government has been involved in taking different initiatives for the development and sustainability of physical education to improve the quality of the students and society (Abdullah et al., 2016). Importantly, across developed nations, physical education is readily pursued to make the general public available with the opportunity to seek reliable learning. In Australia, the government has significantly provided the opportunity for learning physical education to the people because it is believed that a more reliable education would enhance the quality of life of the people effectively (Miftachurochmah and Sukamti, 2022). Oppositely, the people in African countries are not provided with the opportunity for effective physical education; as a result, the American people are left behind in their ability to establish critical thinking (Khaonuan, 2022).

It is essential to understand that innovative tools sometimes create unnecessary stress among teachers. Such a rise in stress level could be because of the perception of increased workload concerning obsolete procedures being pursued in physical education. To be specific, mental workload refers to the stress related to work or any activity in the mind of any individual (Wachs et al., 2019). It is noted that mental workload affects the human brain a lot. Similarly, the intention of the human being is their willingness or desire to do anything and to perform any activity (Shukri et al., 2016). In comparison, the unnecessary increase in thoughts can significantly reduce the chances for an individual to pursue his or her goals, both in personal and professional life. At the same time, this decision-making process is a critical antecedent that an individual mind goes through before achieving the required targets (Tanantong et al., 2022). Likewise, an individual’s self-efficacy has a role in making individuals more productive in terms of their thought processes and utilization of resources for completing the tasks at hand. Individuals with low self-efficacy depend on others to achieve their goals (Lin et al., 2022). Therefore, to let educationists pursue innovation across the physical education system, policymakers will have to increase their level of self-efficacy, followed by effective management of their workload, intentions, and thought processes. As innovation in physical education is to bring new and alternative ways in the development of physical education to improve the quality and standard of life of people, it needs psychological strength and willingness in the form of self-efficacy.

The objective of this study was to identify the role of cognitive factors and self-efficacy in innovation in physical education in the context of China. It is noted that the earlier literature is silent about the critical role of cognitive factors and self-efficacy in innovation in physical education in the context of China (Zhang, 2021). It is observed that physical education plays a critical role in the mental and physical development of people to the advanced level. Notably, in China, it is reasonable to understand that physical education is not provided to the people according to the requirements of the students and community (Sum et al., 2021). It is due to the reason that there is a gap in policy development and moral implications for physical education for children and adults in China (Kosiba et al., 2019). Therefore, because of the above-stated reasons, this study considers the specific compilation of variables (i.e., cognitive factors with self-efficacy) to address the ever-rising concern of scarcity of innovation within physical education in China.

This study is designed to provide significant theoretical and practical implications for the improvement and innovation in physical education in China. It is critical to understand that no earlier study has provided the implications related to the role of cognitive factors and self-efficacy in innovation in physical education in China. Indeed, Chinese people are more concerned, but they are provided with few opportunities for physical education in innovative ways (Kosiba et al., 2019). Education trends have changed in educational institutions, and digital education is provided to Chinese people (Kern and Graber, 2017). Similarly, according to Kern and Graber (2017), the community demands innovations in physical education for better development of understanding for achievement in society. In this regard, this study aims to provide significant theoretical and practical implications related to the role of cognitive factors and self-efficacy in innovation in physical education in the context of China to improve the quality of education and life of the Chinese people at large. Furthermore, this study provides significant and reasonable future directions related to the scope of study that will be helpful for future researchers not to repeat the same work but to consider the unaddressed area in literature for significant contribution.

## Literature review and hypotheses development

**FIGURE 1:**
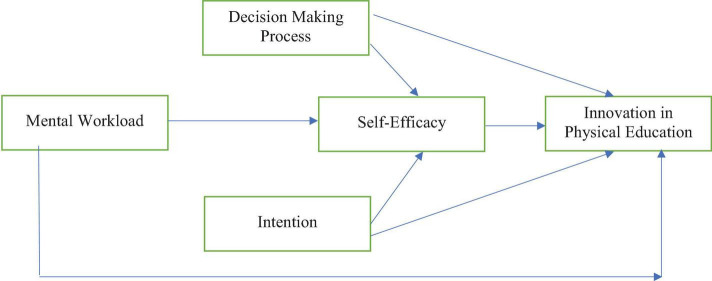
Research model.

The research model of this study is based on the social learning theory, which has a role in explaining the learning-oriented behavior of individuals. In this way, social learning theory emphasizes that people are learning about the importance of observing and modifying their behaviors, emotions, and attitudes (Caspi et al., 2006; Angela, 2014; Hasanah et al., 2022). Moreover, this theory highlights that learning is an art and science developed with a proper social context because it matters a lot in people’s learning (Caspi et al., 2006). Furthermore, the theory also explains that modeling and observational learning are critical for proper development and understanding as it provides a way to succeed through innovative learning (Hasanah et al., 2022). However, during the literature reviews for this study, it was critically analyzed that as far as physical education is concerned, different alternative ways are helpful to provide effective learning within physical education. Therefore, this study is based on the variables that are also contributing toward innovative physical education (see Figure 1). Indeed, for innovatively learning physical education, different cognitive factors matter a lot (Nazari and Alizadeh Oghyanous, 2022). To begin with, this study has opted for a variable that tentatively holds the potential to influence innovative learning and provides aspirants with the opportunity for improved learning behavior. Also, this study has mental workload and decision-making process as the influencing factors that are also contributing to the development of the innovative physical education learning process. Hence, the findings of the study would be of reasonable assistance toward understanding the role of innovatively done physical education in causing improved psychological and intellectual development of the masses.

### Mental workload, self-efficacy, and innovation in physical education

Physical education is vital for any community because it provides information related to human health and the mental ability to perform any activity. It is critical to understand that with the help of physical education, the government of Japan is providing the opportunity to the students to effectively develop their critical learning skills to boost their performance in the long run (Mitrotasios et al., 2022). However, it is also noted that with the help of physical education, the ability to perform different activities increases multi-fold, with the proper state and intensity of mental comfort. Therefore, if individuals are provided with the facility to keep their mental health in check, they will be in a better position to effectively/innovatively implement their physical education plans (Kosiba et al., 2019; Alim et al., 2022). It is the critical responsibility of the management of the schools to provide adequate physical education to the students to furnish their critical learning and abilities to improve their performance. Indeed, people, who are not mentally alert, believe that they might be underperforming because of the given psychological state (Wachs et al., 2019; Vanore et al., 2021).

Moreover, physical education is not only about providing insights related to physical wellbeing but it also allows individuals to learn and develop their cognitive, mental, and critical thinking-related abilities for improved performance outcomes. Notably, the people who have the concept of self-efficacy are more concerned with achieving their goals because they believe that with the help of this critical skill, it would be effective for them to grow and develop strategies for effective learning (e Pina et al., 2021; Lin et al., 2022). Similarly, people with greater self-efficacy remain in a better position to develop sufficient professional skills, which help them perform better at a more comprehensive level. The responsibility of the government and other stakeholders is to provide awareness related to self-efficacy to the people and positively motivate them to enhance their mental ability and critical learning abilities to perform multiple activities effectively (Kekäläinen et al., 2022; Lin et al., 2022). In Nigeria, it is observed that the people are not provided with adequate physical education facilities, which is hindering their abilities to develop critical learning skills in order to become high-performance achievers, while the case is otherwise for people across first world countries (Kekäläinen et al., 2022; Smith et al., 2022).

Moreover, the students who are more concerned about their self-efficacy and are self-motivated to get physical education through innovative means usually believe that such innovative tools would help them enhance their skills (Pina et al., 2021; Lin et al., 2022). In this way, the government’s responsibility is to create awareness and motivation related to the student’s self-efficacy to provide an alternative for productively getting physical education to improve their performance. It is also essential to understand that self-efficacy helps people develop their mental ability if they are provided with the appropriate directions to make them achieve their goals. No doubt, in most cases, people use to have the required motivation and the willingness to achieve their goals to a more considerable extent, but most of the time, they are not provided with the right to develop their critical abilities for practical goals attainment (Presti et al., 2013; Rezaei, 2022). Importantly, if the opportunity is provided to the people and they are educated related to their mental ability, then their cognitive development would be effective, and there would be better equipped to perform certain activities to achieve the desired success effectively. However, it is noted that Chinese students are not provided with innovative physical education because of specific inabilities held by both government and other stakeholders (Kuk and Holst, 2018; Wanaratna et al., 2019). Therefore, it is the fundamental right of Chinese students to get innovative physical education in a different alternative way to develop their learning capacity and improve their mental state for better productivity related to human instinct (Nguyen et al., 2022). Besides men, Chinese female physical education students must also be equipped with the required innovative learning opportunities to prepare better to cater to the opportunities posed by the environment (Lee, 2018).

*H1. There is a relationship between mental workload and self-efficacy.*

*H2. There is a relationship between mental workload and innovation in physical education.*

*H3. There is a relationship between self-efficacy and innovation in physical education.*

### Decision-making process, self-efficacy, and innovation in physical education

It is essential to understand that making the right decisions for more significant achievement is the objective of rational and intellectual people because they used to be more concerned about their long-term orientation. However, it is the responsibility of the people to understand their mental ability and critical skills that are appropriately required for their decision-making, because until and unless they are not provided with appropriate reason for self-improvement, it would be difficult for them to improve their standard of intellectual abilities, required for achieving their goal (Radley et al., 2017; Lee, 2018; IGE et al., 2020; Regier, 2021). It is also noted that the people of China are facing a different kind of crisis concerning physical education as it is not rightly provided to the people to improve their living standards on the advanced level (Xia et al., 2022). It is because the Chinese industry and government look more focused on the practical education of people to improve the industrial sector contributing to the economic performance of the country (Zhang et al., 2019; Zhao et al., 2020). As a result of it, Chinese students lack the opportunity of getting an appropriate level of physical education because the education system is linked with the industry, and more practical teaching related to industrial growth and management is provided to the student (Nazari and Alizadeh Oghyanous, 2022; Qiu et al., 2022; Zeng et al., 2022).

Importantly, it must be considered that practical education is not the sole contributor to people’s success; physical education equally plays an equally critical role in making people prosperous. First, the people of Australia are more rational and intellectual in thinking and decide on their future goals because they believe that if they are not provided with the right opportunity to achieve their goals, then it would be lame for them to get success (Radley et al., 2017; Ghufron and Ermawati, 2018; Nazari and Alizadeh Oghyanous, 2022). Second, the Japanese students are also more independent in their intelligent decisions making, and the parents believe that their kids are self-sufficient for thinking and deciding as they are provided with the appropriate physical education to grow and improve their mental status to remove the mental discount from their life (Ghufron and Ermawati, 2018; OKADA, 2021). Third, the individuals of South Korea are equally capable of their intelligent behavior because they are provided with the proper physical education in the right way to improve their capacity for thinking and decision-making (Sabater-Grande et al., 2022). Therefore, the responsibility of the physical education institutes management is to consider to what extent it would be reasonable and affordable for the government to provide physical education to the students and other people of the society and also to understand that it is critical for the growth of the mental level of the people. Indeed, in different surveys, it is concluded that physical education improves thinking capability because it provides a way of comforting the mind from a different kind of mental crisis and removing the level of stress from the human mind (Nazari and Alizadeh Oghyanous, 2022; Smith et al., 2022; Xu et al., 2022).

In this regard, it is critical to realize that proper physical education has an unmatchable role in facilitating the students to develop their physical and mental abilities to a level where they can continue living respectably and successfully. Moreover, it is also noted that the students with low physical education interaction are not more concerned with their critical thinking ability and their decisions lack intellectual outlook (Peng and Lin, 2019). In India, the students who are getting appropriate and the right physical education in the educational institutes, these students are more intelligent because physical education boosts the human thinking ability to the advanced level if it is provided in the right way (Ghufron and Ermawati, 2018; Hu et al., 2022). Oppositely, those students who are studying in an institute that fail to deliver physical education in innovative ways are not considered intellectual and are left behind in society compared to the intellectual ones (Nazari and Alizadeh Oghyanous, 2022). Furthermore, decision-making is an art that helps people to make the right decision at the right time with the right resources to get the right objective. Importantly, it is a common observation that in their teens, students are required to make important decisions related to their educational and professional goals, so students who have been seeking innovative physical education remain in a better position to make such decisions effectively (Wachs et al., 2019; Clark et al., 2022). Oppositely, the students who are not provided with the right opportunity to develop their mental ability remain unbothered about their critical thinking abilities as they believe they are already critical thinkers without getting the proper physical education and exercise and end up making false decisions. Therefore, it is hypothesized, that:

*H4. There is a relationship between the decision-making process and self-efficacy.*

*H5. There is a relationship between the decision-making process and innovation in physical education.*

### Intention, self-efficacy, and innovation in physical education

One needs to be mindful that developing self-efficacy enables the person to have the identifiable role of having the right intentions. Likewise, the intention has an equally vital role in the physical education of the students to achieve the desired purpose of making them creative intellectual thinkers (Purwanto et al., 2021; Wang et al., 2021). In this regard, the students who are willing to achieve long-term goals in society need to be more concerned about their intensity of intentions and willingness to pursue and achieve the desired goals (Khoi et al., 2018). However, for proper decision-making, it is critical to have the intention because, without intention, no one can think of making intentional, meaningful decisions toward attaining their goals. Indeed, the people who are more concerned about their goals are getting innovative physical education to develop the skills required to think critically and make intellectually sound decisions (Penketh et al., 2014; Fullana and Pallisera, 2019). The responsibility of the stakeholders is to provide physical education to the students in innovative ways to enhance their critical thinking ability to the advanced level to get better outcomes from students. Similarly, the students who are living in Sydney are more concerned about their intentions behind the tentative decisions because they believe that by being well-informed about the ins and outs of their actions, they will be in a better position to achieve the desired outcomes (Watson, 2007; Osorno and Federico, 2022). Moreover, it is also noted that the students in backward countries like Nepal and Bhutan are not provided with the opportunity of getting innovative physical education to develop advanced intellectual skills in order to compete with the students of the modern universities of the developed countries like Australia and Canada (Yu et al., 2018; Blondé et al., 2022; Mitrotasios et al., 2022).

As has already been elaborated, intentions are critical for making the right decisions, and it is also vital that in order to help individuals avoid increased mental workload (i.e., excessive thinking), innovative physical education has a role to play. Similarly, the management of the educational system has an integral part in developing the right intentions of the students by providing the right opportunities related to physical education (Aguillon et al., 2020; Din et al., 2020). Furthermore, the people who are facing different kinds of stress in their life if they are provided with more opportunities to improve their intention and intellectual ability to deal with different kinds of situations, then it would be more feasible for them to achieve the target objective (Throop Robinson et al., 2022; Werner et al., 2022). Likewise, in the American institutes, physical education is considered critical to be provided to the students and the other people in the community because it helps in developing the critical thinking abilities of the student (Regier, 2021; Nazari and Alizadeh Oghyanous, 2022; Osorno and Federico, 2022). The modern trends in education are improving the standard of life of people because the modern generation is required an alternative of learning that are more suitable and understandable by the modern generation.

Modern educational trends are more inclined and focused on improving the living standards of the modern generation. In this regard, the responsibility of educational institutes is to have creativity in physical education and to provide it in the best way to the students and the other people in the community to enhance their critical thinking skills. It is noted that in the educational institute of Portugal, the students are more conscious of innovative facilities provided across physical education institutes because they believe that physical education is equally vital as necessary is the academic one (Akuratiya and Meddage, 2020; Sormunen et al., 2020; Prasetiyo et al., 2022). In the like manner, those students who are not provided with the right opportunities in selecting the creative way of physical education for learning and awareness usually remain uninterested in developing the right abilities and strategies for attaining their professional and life-related goals. It is the responsibility of the management to provide the best alternative ways for developing the critical thinking ability of the people to improve their standard of living to the advanced level by providing them with the physical education equivalent to the educational standards offered across America, Australia, and Canada (Kember et al., 2000; Watson, 2007; Ayon and Islam, 2019; Mitrotasios et al., 2022; Nazari and Alizadeh Oghyanous, 2022). In this way, students will be developed for better, more productive, and critically sound decisions, backed by the required intentions and intensity of the psychological workload. So, it hypothesized that:

*H6. There is a relationship between intention and self-efficacy.*

*H7. There is a relationship between intention and innovation in physical education.*

*H8. There is a mediation role of self-efficacy between the decision-making process and innovation in physical education.*

*H9. There is a mediation role of self-efficacy between the intention process and innovation in physical education.*

## Methodology

For this study, the quantitative data collection method was adopted to collect the data from the target respondents to determine the relationship status between different variables taken in this study. This study considered the survey-based method of data collection as it was considered the most appropriate, economical, and time-saving data collection technique available. Therefore, for this study, the structured questionnaire was prepared on a five-point Likert scale to collect data, as this method is more appropriate for measuring the relationship between the variables in the framework of the study. However, the scale items for this study were taken and adapted from the previous creditable studies. Therefore, the scale items for innovation in physical education were taken from a study by Kern and Graber (2017).

Moreover, the scale items for intention were taken from the study of Sarlan et al. (2012) to measure the relationship between intention and innovation in physical education. Also, the scale items for self-efficacy were taken from the study of Sagone and De Caroli (2014) to measure the role of self-efficacy in intention and the innovation of physical education. Further, the scale items for the decision-making process were taken from the study of Donelan et al. (2016) to measure the decision-making process concerning innovation in physical education. Similarly, the scale items for mental workload were taken from the study of Ramos-Villagrasa et al. (2019) to measure the relationship between mental workload and self-efficacy. Furthermore, these scale items were revised three times by the experts, and with positive expert opinions, these scale items were considered to collect the data from the respondents for this study.

In this study, the data were collected from the students of China who are getting physical education. The target respondents of this study were Chinese students because this study was conducted in the context of physical education in China. Notably, 800 questionnaires were provided to the respondents, but in return, only 420 questionnaires were returned, and the expected response rate was 40%. Further, 40 questionnaires were eliminated from it because of incorrect information, and only 380 responses were considered to collect the data from the target respondents.

## Findings and results

In Table 1, the results related to confirmatory factors analysis are presented. This test was conducted to identify the factor loadings values for the constructs used in this study for each variable presented in the theoretical framework. In this regard, the confirmatory factor analysis test was conducted to find these values for the test of reliability and validity of the constructs used in this study. According to the results, the values of factor loadings for each construct must not be lesser than 0.40 recommended by Wong (2013).

**TABLE 1:** Results of confirmatory factor analysis.

Variables	Constructs		Loadings
Mental workload	MW1	I managed to plan my work so that I finished it on time	0.520
	MW2	I kept in mind the work results I needed to achieve	0.491
	MW3	I was able to carry out my work efficiently	0.877
	MW4	I took on extra responsibilities	0.471
Intention	IN1	Getting physical education makes me comfortable	0.926
	IN2	I have avoided physical education innovation	0.917
	IN3	Working with a trainer makes me comfortable	0.859
Self-efficacy	SE1	I managed to achieve a fixed objective	0.805
	SE2	I manage a difficult situation	0.466
	SE3	I decide with the risk to fail	0.457
	SE4	React adequately facing a failure	0.484
Innovation in physical education	IPE1	I accept new ideas in physical education	0.715
	IPE2	I want physical education as inventive and creative	0.662
	IPE3	I enjoy new methods of physical education	0.618
Decision making process	DMP1	My decision-making is knowledge-based	0.656
	DMP2	I consider uncertainty and unknowns in my decision-making approach	0.817
	DMP3	My decision-making is consistent	0.794

MW, mental workload; IN, intention; SE, self-efficacy; IPE, innovation in physical education; DMP, decision-making process.

### Measurement model

**FIGURE 2:**
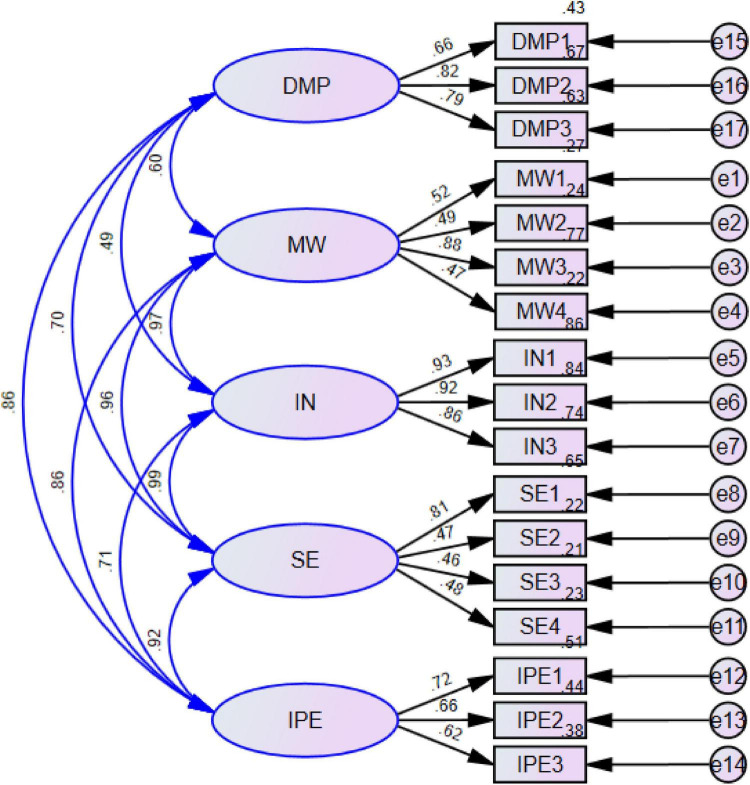
Measurement model. MW, mental workload; IN, intention; SE, self-efficacy; IPE, innovation in physical education; DMP, decision-making process.

In this study, the AMOS data analysis software was used to identify values for validity and reliability of each construct, and the results are shown in Table 2. Moreover, according to the results in Figure 2, there is a clear correlation, reliability, and validity. In this regard, all the values of composite reliability (CR) were more significant than 0.70, as Hair et al. (2017) recommended. Furthermore, all the values of average variance extracted (AVE) were more significant than 0.60, as Wong (2013) recommended. Therefore, the results reveal apparent validity and reliability between the constructs used in this study to collect the data from the respondents.

**TABLE 2:** Validity analysis.

	CR	AVE	MSV	MaxR (H)	DMP	MW	IN	SE	IPE
DMP	0.801	0.676	0.733	0.817	**0.759**				
MW	0.890	0.676	0.939	0.812	0.600***	**0.713**			
IN	0.928	0.612	0.986	0.934	0.490***	0.969***	**0.801**		
SE	0.843	0.626	0.986	0.728	0.698***	0.962***	0.993***	0.771	
IPE	0.705	0.644	0.837	0.710	0.856***	0.859***	0.715***	0.915***	**0.866**

MW, mental workload; IN, intention; SE, self-efficacy; IPE, innovation in physical education; DMP, decision-making process. The bold values indicate the results for the corresponding statistics for the whole variable, not the items.

***Denotes significance at 1%.

In the same way, the measurement model fit was checked by analyzing and evaluating the root mean square of approximation, absolute fit measures, standardized root mean square residual, comparative fit index, normed fit index, and adjusted goodness of fit, which is presented in Table 3. Furthermore, the recommended threshold was achieved for all the values and tests for this model.

**TABLE 3:** Confirmatory factor analysis model.

Measure	Recommended threshold	Abbr.	Scores
Chi-square/df (CMIN/DF)	<3.0	2/df	2.0018
Comparative fit index	>0.90	CFI	0.81
The normed fit index	>0.90	NFI	0.78
Goodness of fit	>0.90	GFI	0.85
Adjusted goodness of fit	>0.80	AGFI	0.79
Root mean square residual	<0.08	RMR	0.08
Standardized root mean square residual	<0.08	SRMR	0.08
Root mean-square error of approximation	<0.08	RMSEA	0.08

Lastly, to test the discriminant validity for the constructs of each scale item of this study, the Heterotrait–Monotrait (HTMT) method was considered suitable. In this way, AMOS was used to verify the values of HTMT to determine the discrimination between the scale items used for each study variable. According to the results shown in Table 4, all the values of discriminant validity achieved were within the recommended threshold of 0.90 recommended by Henseler and Fassott (2010).

**TABLE 4:** Discriminant validity—Heterotrait-Monotrait (HTMT).

	DMP	MW	IN	SE	IPE
DMP					
MW	0.807				
IN	0.512	0.750			
SE	0.843	0.728	0.769		
IPE	0.734	0.709	0.756	0.755	

MW, mental workload; IN, intention; SE, self-efficacy; IPE, innovation in physical education; DMP, decision-making process. The shaded cells are the main results.

### Structural model

The results of the hypotheses are presented in Table 5 of the study. H1 was tested to check the mental workload, which has a significant effect on self-efficacy (β = 0.421, *t* = 3.723, *p* = 0.000), and H1 is accepted. H2 was tested to check its significance and according to the results, mental workload has a significant effect on innovation in physical education (β = 0.212, *t* = 3.721, *p* = 0.000), and H2 is accepted. H3 was tested to check its significance, and according to the results, self-efficacy has a significant effect on innovation in physical education (β = 0.475, *t* = 3.689, *p* = 0.000), and H3 is accepted. H4 was tested to check its significance, and according to the results, decision-making process has a significant effect on self-efficacy (β = 0.267, *t* = 3.912, *p* = 0.000), and H4 is accepted. H5 was tested to check its significance, and according to the results, the decision-making process has a significant effect on innovation in physical education (β = 0.442, *t* = 4.413, *p* = 0.000), and H5 is accepted. H6 was tested, and according to the results, intention has a significant effect on self-efficacy (β = 0.459, *t* = 3.921, *p* = 0.000), and H6 is accepted. H7 was tested, and according to the results, intention has a significant effect on innovation in physical education (β = 0.252, *t* = 4.712, *p* = 0.000), and H7 is accepted. H8 was tested to check its significance, and according to the results, there is a significant mediating role of self-efficacy between the decision-making process and innovation in physical education (β = 0.432, *t* = 4.627, *p* = 0.000). Furthermore, H8 is accepted. Lastly, H9 was tested, and the results highlighted a mediating role of self-efficacy between the relationship of intention and innovation in physical education (β = 0.098, *t* = 3.721, *p* = 0.000), and H9 is accepted.

**TABLE 5:** Standardized path coefficient.

Hypotheses	Relationship	Beta	*t*-values	*p*-values	Decision
H1	Direct	0.421	3.723	0.000	Accepted
H2	Direct	0.212	3.721	0.000	Accepted
H3	Direct	0.475	3.689	0.000	Accepted
H4	Direct	0.267	3.912	0.000	Accepted
H5	Direct	0.442	4.413	0.000	Accepted
H6	Direct	0.459	3.921	0.000	Accepted
H7	Direct	0.252	4.712	0.000	Accepted
H8	Mediation	0.432	4.627	0.000	Accepted
H9	Mediation	0.098	3.721	0.000	Accepted

## Discussion

H1 and H2 results reveal a significant relationship between mental workload, self-efficacy, and innovation in physical education. The mental workload is stress found in the brain of people working in different organizations to achieve different goals, as highlighted by Lin et al. (2022). The responsibility of the stakeholders of the society and the government is to design the time frame critical for developing critical skills for the people to improve their performance and standard of understanding (Prasetiyo et al., 2022). It is noted that with the help of physical education, individuals can be provided with newer ways of physical exercises, which could help them further improve their critical thinking abilities and can help in reducing their mental exertion (Bush et al., 2022). Similarly, with the help of self-efficacy, human abilities can be improved productively and help them become better off by having the necessary skills required to cope with prevailing problems (Osorno and Federico, 2022). In this manner, innovation in physical education is critical for developing strategies for the benefit of human beings. Further, H3 results reveal a significant relationship between self-efficacy and innovation in physical education. It is critical to determine that self-efficacy is one of the critical success factors that play an essential role in the development and understanding of human personality (Abdullah et al., 2016). It is because, with appropriate goals set for achievement, self-efficacy can provide the best alternative ways and motivation from innate abilities to achieve these goals effectively. People with high self-efficacy are more motivated and more concerned about their values and understanding to achieve their goals and get success in life (Prewett and Whitney, 2021).

Moreover, H4 and H5 results reveal a significant relationship between the decision-making process, self-efficacy, and innovation in physical education. Similarly, it is reasonable to understand that decision-making ability is one of the human beings’ fundamental and unique abilities (Peng and Lin, 2019; Tanantong et al., 2022). It is because human beings are more concerned about their values and priorities to make decisions effectively. However, the people who are more critical thinkers and more intellectual in their understanding, it is easy for them to make decisions in different critical situations according to their mental level (Nazari and Alizadeh Oghyanous, 2022; Rezaei, 2022). Moreover, people with less critical thinking ability are not concerned about making the right decisions because they do not have the innate ability to deal with such issues (Presti et al., 2013; Ghufron and Ermawati, 2018). Importantly, it must be understood that with the help of physical education, the critical thinking ability of people is developed as they are more concerned about their values and want to be at peace with their brains. In this regard, the management must consider the critical role of physical education and innovation in the process of physical education to improve the understanding of the people for making the right decisions at the right time (Kosiba et al., 2019; Alim et al., 2022). In this way, not only the critical thinking ability of the people would be developed but also it would be more comprehensive to make the right decision through a comfortable and sustainable mental level.

Additionally, H6 and H7 results uncover that there is a significant relationship between intention, self-efficacy, and innovation in physical education. It is a fact that the people living in different countries are more concerned about their physical health and the education related to health activities (Dos Santos, 2021; Blondé et al., 2022). It is because people want to achieve sustainable goals with the help of innovation and sustainability, as it is believed that intention is one of the critical success factors behind human psychology and actions. In this way, people with a high level of willingness and intention are more motivated to achieve self-efficacy in an effective way to get life changes. Moreover, people with weak mental ability and very little knowledge of intention related to human dignity are usually less concerned about achieving the right and sustainable goals for effective development (Banman, 2018; Yu et al., 2018; Stephen et al., 2022). In this regard, it is critical to understand that people must be provided with the opportunities to develop their critical thinking ability and intention to achieve their goals sustainably and reliably according to their intention (Langford et al., 2018; Elrayah, 2022). Therefore, by adopting sustainable, innovative ways, people would be better able to achieve their goals productively and effectively (Su et al., 2022).

Notably, the results of H8 uncover a significant mediation of self-efficacy between the decision-making process and innovation in physical education. In this manner, it is critical to understand that people with high thinking ability are more concerned to get innovation in physical education as it is considered appropriate for the improved living standard of the people (Donnelly et al., 2016; Radley et al., 2017; Kuk and Holst, 2018). However, if the people of any community are willing to achieve their goals in a sustainable way to get success, then it would be more appropriate for them to get success through a high level of self-efficacy (Presti et al., 2013; Rahim et al., 2021; Stephen et al., 2022). Similarly, this study highlights that if people are willing to initiate any decision, then their level of self-efficacy provides them with reliable assistance toward achieving success. Lastly, the results of H9 unveil a significant mediation of self-efficacy between the relationship of intention and innovation in physical education. In this regard, it is critical to understand that people with high self-efficacy usually are more focused on attaining their goals by adopting innovative learning tools available (Regier, 2021; Miftachurochmah and Sukamti, 2022; Nazari and Alizadeh Oghyanous, 2022). Therefore, it is crucial for practitioners and researchers probing the human motivations and intentions toward goals attainment to keep a close eye on their level of self-efficacy as an important indicator of success (Sagone and De Caroli, 2014; Regier, 2021; Bush et al., 2022; Elrayah, 2022; Lin et al., 2022). Furthermore, the concept at hand will assist in achieving sustainable development goals, which are essential for people to live productive life at large (Presti et al., 2013; Nazari and Alizadeh Oghyanous, 2022).

## Conclusion

This study was designed to determine the role of cognitive factors and self-efficacy in the innovation of physical education in China. In this regard, it is essential to understand that the study provides a detailed inside of the relationship of the variables taken in the research framework of the study to measure the relationship of cognitive factors in the innovation of physical education. The study highlights a critical role of self-efficacy in the invention of physical education because people with high self-efficacy are more motivated to consider innovative things in their learning and improve their living standards. However, it is also noted that if people do not have self-efficacy and willingness to change the traditional ways of teaching, it would be difficult for them to bring innovation to physical education. Second, this study highlights the critical role of the decision-making process in the innovation of physical education. Indeed, the decision-making process is a cognitive factor to some extent because it is always identified in the human brain. Therefore, the practical and reliable decision-making process provides a guideline to innovate and adopt new ways of learning and application within physical education in China. Oppositely, the people who lack the ability of critical decision-making, these peoples are not actively participating in the innovation of physical education because they lack critical thinking and intellectual abilities.

Further, this study concludes that for innovation in physical education, there is a critical role of intention because intention is one of the critical success factors contributing as a cognitive factor for an individual to initiate an action. In this manner, if the people of China or any community intend to bring changes in the society or the educational system, then it would be a better justification to bring changes in the concerned sector. Similarly, it is also noted that the people with high intentions to bring innovation in physical education are the ones who develop critical thinking abilities for better outcomes. Lastly, it is the prime responsibility of the government and the institution concerned to introduce appropriate innovative physical learning techniques to make the aspirants develop themselves effectively. Such individual intellectual development of individuals will ultimately contribute to the intellectual development of society at large.

## Implications

### Theoretical implications

This study is designed to provide significant theoretical implications for contributing to theory and literature. The findings of this study are significant because it emphasizes that the mental workload, intention, and decision-making process influence the self-efficacy of people, particularly students. Indeed, self-efficacy and determination motivate people to succeed in achieving their goals if they are appropriately getting physical education in innovative ways. The nations that do not focus on innovation in physical education limit the productivity of the people by affecting the mental capability and intellectual abilities required for learning and performing different activities in society. Further, this study also highlights that intention, decision-making process, and self-efficacy positively impact physical education learning methods. The study contributes to the theory as the variables discussed in the context of social learning theory were not discussed by any earlier study up to the researcher’s knowledge. In this manner, this study highlights that the mediating role of self-efficacy must be considered because without a sufficient level of self-efficacy, it will not be easy for individuals to innovate. Therefore, the mediating role of self-efficacy between the relationship between the decision-making process and innovation in physical education and the relationship between intention and innovation in physical education must be considered adequate for future studies to bring innovation to physical education. Importantly, this study provides a robust framework of the research that would be reliable and useful for future studies to consider for developing new dimensions to discover further cognitive factors useful for innovation in physical education. In this way, this study is significant and provides reasonable future directions that are also important to be considered for future studies.

### Practical and managerial implications

The study offers several managerial implications and practical future directions for considering the cognitive factors, which are critical for introducing innovation across the physical education spectrum of China. First, critical thinking ability seminars and sessions must be conducted for the Chinese people to provide them with the factual information required for developing intellectual thinking. It is because until and unless authentic to life, information related to critical thinking would not be provided to the people, and it would be challenging to determine the relationship between cognitive factors and innovation. When the people are appropriately trained for innovation and bringing new ideas in the replacement of the traditional ideas, then more productive and constructive development would be the goal of the people. Second, the results of this study must be considered by the stakeholders of the society and the government of China to observe these variations in a new and constructive way to develop policies that would be effective and reliable in bringing innovations to physical education.

Moreover, the people of any community are more concerned about innovation and bringing new ideas to life. In this way, the people’s focus should be on using the critical success factors and cognitive factors to introduce innovative changes in their overall education canvas. Therefore, with the help of procedural innovation in physical education, the Chinese people can take their skills and critical thinking to the advanced level for effective and reliable living standards in an intellectual way.

## Limitations and future directions

This study aimed to understand the role of cognitive factors and self-efficacy in innovation in physical education in the context of China. In this way, this study is based on the social learning theory by considering intention, decision-making process, and mental workload to check their influence on innovative physical education. In this way, the literature review also explained that multiple alternative factors also influence the role of cognitive factors and self-efficacy in innovation in physical education in China. Therefore, future studies must consider the role of normative beliefs to determine the role of cognitive factors in innovation in physical education for the people of China. Similarly, future studies need to consider the role of perceived norms in the cognitive learning and innovation of physical education in China. Importantly, future researchers need to focus on adaptive behavior to understand the cognitive factors in the relevance of this study.

## Data availability statement

The original contributions presented in the study are included in the article/supplementary material, further inquiries can be directed to the corresponding author.

## Ethics statement

The studies involving human participants were reviewed and approved by Zhengzhou University, China. The patients/participants provided their written informed consent to participate in this study. The study was conducted in accordance with the Declaration of Helsinki.

## Author contributions

SL conceived and designed the concept. RX collected the data. ZZ wrote the manuscript. All authors read and agreed to the published version of the manuscript.
